# Super-Resolution Single Molecule FISH at the *Drosophila* Neuromuscular Junction

**DOI:** 10.1007/978-1-4939-7213-5_10

**Published:** 2017-05-30

**Authors:** Joshua S. Titlow, Lu Yang, Richard M. Parton, Ana Palanca, Ilan Davis

**Affiliations:** Department of Biochemistry, University of Oxford, South Parks Road, Oxford, OX1 3QU UK

**Keywords:** smFISH, Single molecule fluorescence in situ hybridization, Structured Illumination, Super-resolution imaging, 3D-SIM, *Drosophila melanogaster*, Larval neuromuscular junction, mRNA localization, Synapse

## Abstract

The lack of an effective, simple, and highly sensitive protocol for fluorescent in situ hybridization (FISH) at the *Drosophila* larval neuromuscular junction (NMJ) has hampered the study of mRNA biology. Here, we describe our modified single molecule FISH (smFISH) methods that work well in whole mount *Drosophila* NMJ preparations to quantify primary transcription and count individual cytoplasmic mRNA molecules in specimens while maintaining ultrastructural preservation. The smFISH method is suitable for high-throughput sample processing and 3D image acquisition using any conventional microscopy imaging modality and is compatible with the use of antibody colabeling and transgenic fluorescent protein tags in axons, glia, synapses, and muscle cells. These attributes make the method particularly amenable to super-resolution imaging. With 3D Structured Illumination Microscopy (3D-SIM), which increases spatial resolution by a factor of 2 in X, Y, and Z, we acquire super-resolution information about the distribution of single molecules of mRNA in relation to covisualized synaptic and cellular structures. Finally, we demonstrate the use of commercial and open source software for the quality control of single transcript expression analysis, 3D-SIM data acquisition and reconstruction as well as image archiving management and presentation. Our methods now allow the detailed mechanistic and functional analysis of sparse as well as abundant mRNAs at the NMJ in their appropriate cellular context.

## Introduction

In situ hybridization has been a mainstaySingle molecule of cell and developmental biology for determining where and when genes are expressed in wild-type or mutant cells and tissues. The recent development of single molecule fluorescent in situ hybridization (smFISH) methods have increased the sensitivity, ease of application of FISH methodology, and enabled multiplexing with antibodies against specific proteins [1–3]. This next generation FISH approach uses approximately 50 short, fluorochrome-labeled DNA oligonucleotide (oligos) probes, which are approximately 20 bp in length. Such tiled oligonucleotides sets are designed to bind to nonoverlapping regions of a transcript. The large number of probes means that the technique is sensitive enough to detect the majority of individual mRNA molecules in a tissue, achieving a very high signal–noise ratio. The detected individual transcripts appear as bright foci and any off-target labeling by individual oligonucleotides appears as dim, diffuse signal, or low-intensity punctae [1]. Using shorter probes also provides better tissue penetration and enables less harsh hybridization conditions, maintaining antigenicity for Antibody staining and making the technique especially suitable for whole mounted tissues.

The study of RNA biology in neuroscience has been held back by the lack of suitable methods for high quality in situ hybridization in some key experimental models and tissues. *Drosophila* in particular is an excellent model system for elucidating molecular mechanisms of neuronalNeurons development and function in all parts of the nervous system [4–6]. One of the key models for studying synapticSynapse plasticity and physiology is the Larval neuromuscular junction (NMJ) preparation of the body wall musculature. This system also has tremendous potential for studying the role of RNA metabolism in plasticity and physiology [7, 8]. However, while smFISH has been used successfully in *Drosophila* Oocytes and Embryo [9, 10], only traditional RNA FISH methods have been used in the NMJ [11–13]. Such methods have not been widely adopted due to variability, poor signal–noise ratios, and limited sensitivity for sparse transcript expression. Here, we describe our modified smFISH protocol for visualizing single mRNA molecules in the larval NMJ together with endogenous fluorescent proteins and antibody markers. To complement the single transcript sensitivity of smFISH, we used 3D structured illumination microscopy (3D-SIM), a super resolution imaging technique that provides enhanced spatial information regarding the RNA’s subcellular environment [14]. The increased optical resolution of methods like 3D-SIM [15] provide a more accurate representation of whether a transcript resides in or is adjacent to a particular RNP granule or subcellular compartment (*see* **Note** **1**). Furthermore, the relatively mild hybridization and wash conditions required for smFISH allow tissue morphology to be well preserved for meaningful biological interpretations.

Resolving individual transcripts in an intact tissue is extremely powerful for investigating gene expression and mRNA localization. To fully realize the benefits of single transcript detection, an automated quantification workflow saves time and reduces variability. Various computer programs have been developed to automate segmentation and quantification of the numberSingle molecule of foci in an image. We used FindFoci, an open source ImageJ (Fiji) plugin that is part of the GDSC suite [16]. We also used an open source MatLab program called FISHQuant that allows automated segmentation and fluorescence intensity calculations [17], and a user-friendly commercial solution, namely the spot counting algorithm in Imaris. We found that all three programmes performed similarly with our in situ data in automated Quantitation of transcript numbers. To quality control acquisition of raw 3D-SIM data and the 3D-SIM3D Structured Illumination Microscopy (3D-SIM) reconstructions we used the ImageJ (Fiji) plugin SIMcheck [18]. Finally, we managed the relatively large number and size of image files with OMERO and created summary figures with OMERO-Figure, a platform that enables public distribution of the raw image filesSingle molecule.

## Materials

### smFISH Probes

DNA oligonucleotide probes (Stellaris^®^ RNA FISH) were purchased from LGC BioSearch Technologies (California, USA) and sequences were selected using the company’s online probe designer. Alternatively, one could design 30–50 3′ primary amine labeled 18-mer DNA oligonucleotides that cover a region of the chosen gene, order a plate of HPLC-purified oligonucleotides (available from most manufacturers who synthesize PCR primers) and conjugate fluorochromes to the probes oneself [3]. Probes described here were labeled by the manufacturer with either Quasar 570 or Quasar 670 dyes, as using orange and red emitters minimizes background from autofluorescence in the NMJ.

### Larva Neuromuscular Junction Dissection

1. Dissecting microscope with light source.
2. 35 mm petri dish.
3. Sylgard or similar elastomer [19].
4. Insect pins.
5. Microdissection scissors and forceps.
6. Saline buffer: 70 mM NaCl,5 mM KCl, 20 mM MgCl_2,_ 10 mM NaHCO_3,_ 5 mM trehalose, sucrose 115 mM, and 5 mM HEPES, pH 7.2.

### Fixation and Hybridization (See **Note****2**)

1. Fix solution: PBS, 0.3%Triton X, 4% formaldehyde from freshly thawed aliquots of 16% EM grade PFA.
2. PBTX: PBS, 0.3%Triton X.
3. Bovine serum albumin (nuclease-free).
4. 70% ethanol.
5. Wash buffer: 10% 20× SSC (3 M NaCl, 0.3 M sodium citrate, pH 7.0), 10% freshly thawed deionized formamide, 80%DEPC-treated water.
6. Hybridization buffer: 10 w/v % dextran, 250 nM smFISH probe in Wash buffer.
7. (Optional) for immunohistochemistry: appropriate primary and secondary antibodies diluted in Wash buffer.
8. (Optional) to counterstain axon terminals: dye conjugated anti-horseradish peroxidase antibodySingle molecule diluted 1:100 in Wash buffer.
9. (Optional) to counterstain nuclei: 1 μg/mL DAPI in Wash buffer.

### Mounting

1. Glass slide and glass coverslips (High Precision No. 1.5 coverslips for 3D-SIM).
2. Double-sided adhesive tape.
3. Vectashield mounting medium.
4. 100 nm Tetraspek beads.

### Image Acquisition

1. For conventional imaging: wide-field epifluorescence microscope, spinning disk or laser scanningSingle molecule confocal microscope with a 60× or 100× 1.3–1.4NA oil or silicone oil immersion objective. We used an Ultra- VIEW VoX from PerkinElmer mounted on an IX81 Olympus microscope with 60× 1.35 NA oil immersion objective and an electron-multiplying charge-coupled device camera (ImagEM; Hamamatsu Photonics).
2. For 3D-SIM microscopy: images are acquired on a DeltaVision OMX, V3-Blaze (GE) with 60× 1.3 NA silicone oil immersion objective from Olympus (*see* **Note** **3**).

### Image Processing and Analysis

1. ImageJ/FIJI with SIMcheck and FindSpot plug-ins.
2. fairSIM [24].
3. OMERO server (https://www.openmicroscopy.org/site/support/omero5.2/sysadmins/unix/server-installation.html).
4. (Optional) Matlab with the FISHQuant script.
5. (Optional) Imaris.

## Methods

### Larva Neuromuscular Junction Dissection

1. Video protocols for *Drosophila* larva dissection are available online [20, 21]. Pin the larva dorsal side up on a 35 mm Petri dish filled half way with Sylgard, by placing pins at the anterior and posterior ends.
2. Cover the larva with a few drops of saline buffer.
3. Use microdissection scissors to create a small incision at the centre of the dorsal midline.
4. Extend the incision along the dorsal midline toward the posterior end, then from the centre towards the anterior end of the larva, make the cuts as superficial as possible so as not to damage the underlying nervous system and muscle tissues.
5. Carefully remove gut tissue by holding the trachea with forceps and cutting the tracheal attachments at each abdominal segment. After cutting the trachea on either side the gut tissue and other organs can be carefully removed all at once, leaving the brain and nerves intact.
6. Place two pins into the outer “shoulders” of the anterior body wall and gently stretch the tissue away from the midline. Do the same for the posterior side.
7. At this point the brain can either be removed, by cutting the nerves just above the muscle tissueSingle molecule, or properly positioned for in situ imaging by gently stretching the head pin.

### Fixation

1. Replace the dissection buffer with fix solution and incubate by gentle rocking at room temperature for 25 min.
2. Remove the fix buffer and rinse 3× with PBTX.
3. (Optional) If immunohistochemistry is to be performed, block the tissue by incubating for 60 min in PBTXSingle molecule with 1% RNAse free bovine serum albumin.
4. Carefully transfer the tissue to a 0.75 mL microcentrifuge tube filled with 0.2 mL 70% ice-cold ethanol and incubate for 4–24 h at 4 °C.

### Hybridization

1. Replace the ethanol with 0.2 mL wash buffer and incubate for 10 min at 38 °C with gentle rocking.
2. Replace the wash buffer with 0.1 mL hybridization buffer and incubate for at least 4 h (ideally overnight) at 38 °C with gentle rocking.

### Washing and Counterstain

1. Remove the hybridization buffer and rinse 3× with 0.2 mL wash buffer.
2. Incubate the tissue in 0.2 mL wash buffer for 45 min at 38 °C with gentle rocking.
3. (Optional) For counterstaining, add secondary antibodies (1:500 dilution) and/or DAPI. To label Axon terminals in the NMJ use dye conjugated anti-horseradish peroxidase antibody (1:100 dilution) (*see* **Note** **4**).
4. (Optional) If tissues are counterstained, remove excess dye by washing 3× in the wash solution and incubating at room temperature for 15 min with gentle rocking.

### Mounting

1. Remove the wash buffer and incubate tissue in Vectashield for several minutes (*see* **Note** **5**).
2. Place thin strips of double-sided tape across a glass microscope slide spaced about as wide as the coverslip.
3. Place a drop (~30 μL) of Vectashield at the centre of the slide, between the strips of tape.
4. Position the tissues dorsal side up in the Vectashield and carefully place a coverslip on the strips of tape (*see* **Note** **6**).
5. Seal the coverslip with multiple layers of clear nail varnish, taking care not to let the varnish come in contact with the Vectashield.
6. For super-resolution imaging with silicone immersion lens (NA 1.3): dilute Vectashield to 70% with water and use High Precision No. 1.5 coverslips.

### Image Acquisition on the Spinning Disk Confocal Microscope

**Fig. 1:**
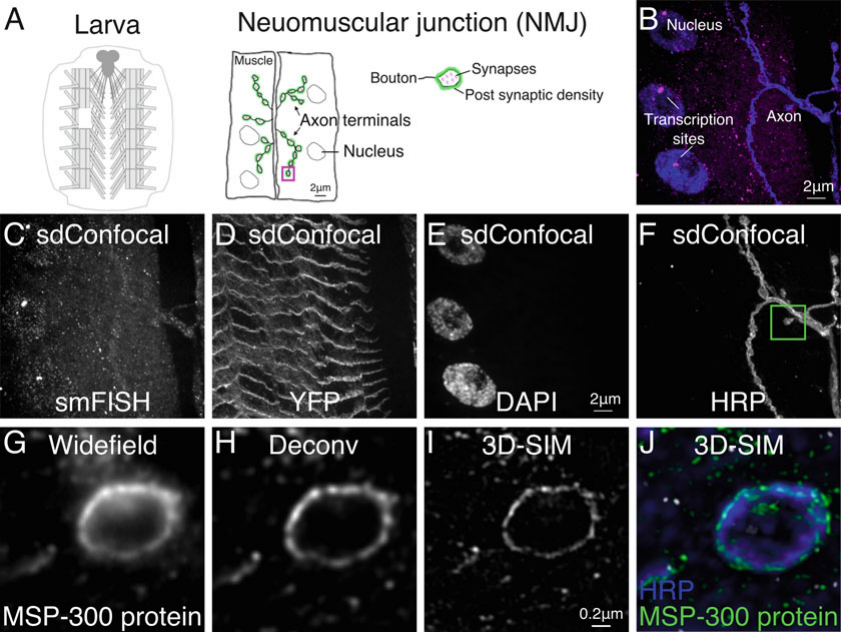
Example of smFISH data acquired from the larva neuromuscular junction (NMJ) with spinning disk confocal (sdConfocal) or 3D Structured illumination microscopy (3D-SIM3D Structured Illumination Microscopy (3D-SIM)). (**a**) Schematic of the larva fillet preparation indicating the location of an NMJ and the major subcellular compartments. (**b**) Merged 3D projection of a specimen labeled with smFISH probe (*magenta*) and Alexa 647-conjugated anti-HRP counterstain (*blue*). Image was acquired on a spinning disk confocal with 60× 1.35 NA oil objective. (**c**) sdConfocal image of MSP-300-smFISH: a coding region of MSP-300 mRNA was hybridized with a probe set containing 48 short oligos (18 nts) individually labeled with Quasar 570. (**d**) The MSP-300::YFP fusion protein is easily detected in the smFISH preparation. Nuclei and NMJ Axons were labeled with DAPI and Alexa 647-conjugated anti-HRP respectively (**e, f**). Box in **f** shows the relative region of a bouton, (*see* **g–j**). Enhancement in resolution can be seen by comparing widefield (**g**) and deconvolved (**h**) images of the MSP-300 label to 3D-SIM reconstructions (**i, j**)

1. Acquire optical sectionsSingle molecule of the region of interest using optimal imaging configuration for your system, i.e., choosing appropriate beam splitter, emission filter, laser power, and pixel size.
2. Exposure times of 600–800 ms are often required for camera-based imaging systems. The slowest scan speed and line averaging are often necessary on scanning confocal systems.
3. Single transcripts generally appear as discrete punctae with consistent intensities. An exception is in the nucleus where a high concentration of nascent transcripts form a much larger and brighter fluorescent focus at the gene locus (Fig. 1b).

### Image Acquisition for 3D-SIM3D Structured Illumination Microscopy (3D-SIM)

1. Acquire 3D-SIM dataSingle molecule according to manufacturer’s guidelines and good imaging practices, balancing signal–noise and bleaching while correcting for spherical aberration [22, 23]. Check raw data with the open source ImageJ plugin SIMcheck (Fig. 2).

**Fig. 2:**
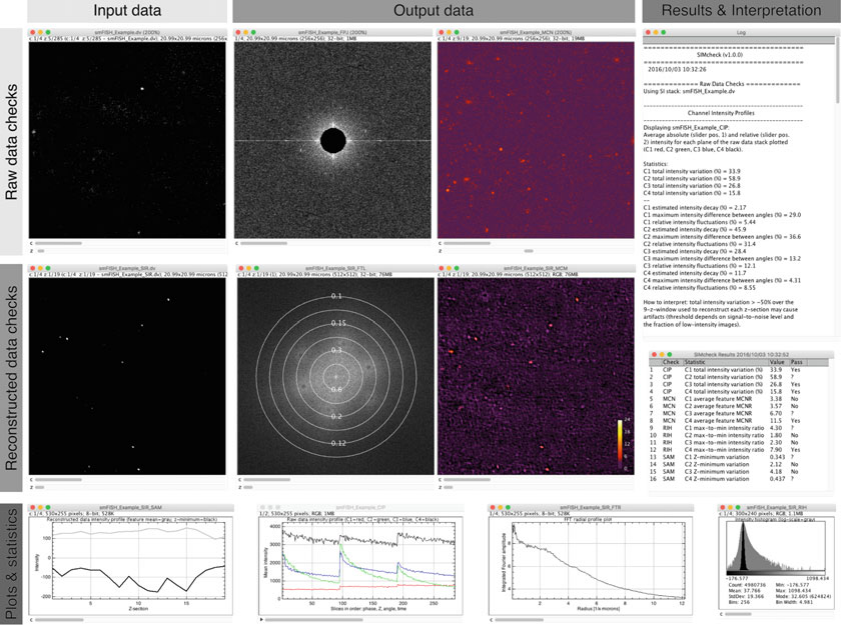
Representative output from SIMcheck. For detailed explanation of these plots and statistics *see* [18]
2. Perform image reconstruction using commercial software that accompanies the instrument (in our case, SoftWORX by GE for the OMX V3) or an open-source alternative, such as fairSIM [24]. For multichannel imaging you will need to register channels using Tetraspec beads data and an appropriate Image registration software.
3. Check the quality of reconstruction using SIMcheck (Fig. 2).

### Image Management Using the OMERO Database (Open Microscopy Environment)

1. Install an OMERO server and import your data to it with the OMERO.insight client software [25–27]. Use the “tagging” facilities to organize your imaging data. The initial installation and subsequent super-user management of the server requires some degree of system administration experience. OMERO software is open source and released by the OME Consortium at www.openmicroscopy.org. Look at the online video tutorials, such as http://help.openmicroscopy.org/importing-data-5.html and at the installation instructions. This client-server software integrates visualization, data mining, and image analysis of biological microscopy images. OMERO through its use of the Bio-Formats importer (http://www.openmicroscopy.org/site/products/bio-formats) and conversion to OME-TIF supports over 140 image file formats and the raw data can be managed from the web or exported from the online platform to a third party software like ImageJ (Fiji).
2. Use the OMERO web browser to view and organize the primary imaging data using searchable tags.
3. Use OMERO to share the data between collaborating scientists from any location with Internet access.
4. Create and highlight figures from typical data sets using OMERO.figure (video http://figure.openmicroscopy.org/videos.html). OMERO.figure uses unique OMERO IDs for each image, from which the figure panels are made, to link to the originalSingle molecule raw image data. Therefore, figures can be adjusted with great ease and other scientists in a team can easily view the original data. While OMERO-Figure can be used to add some annotations to the figure panels, we find that publication ready figures require the use of other image manipulation software.

### Image Analysis (See **Note****7**)

#### Find Foci

**Fig. 3:**
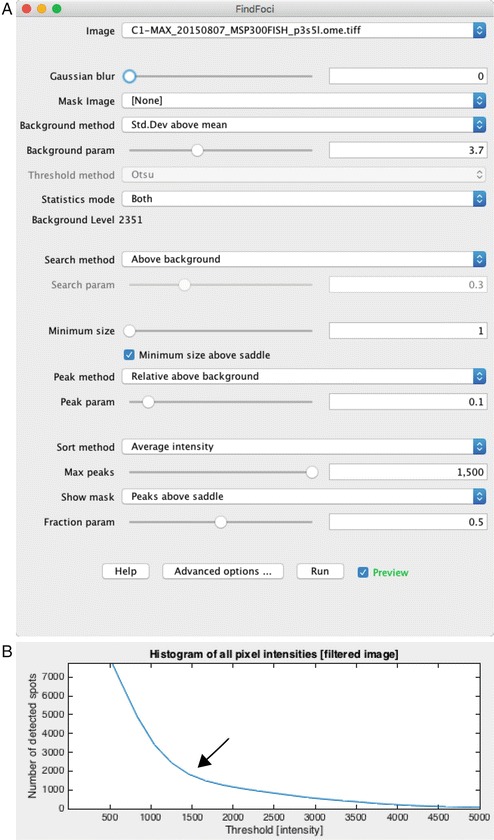
Thresholding parameters for counting spots in the FindFoci ImageJ plugin. With these settings (**a**) the “Background param” slider is adjusted until all spots are identified in the image. The FISHQuant and Imaris Spots applications use intensity thresholding (**b**), which is used to provide an initial separation between background and high intensity spots (*arrow*), which are then refined using additional parameters

1. Install and open the FindFoci GUI application in ImageJ; Plugins >GDSC > FindFoci > FindFocus GUI [16].
2. Open an image in ImageJ and splitSingle molecule the channels; Image > Color > Split Channels.
3. Select the smFISH channel in the FindFoci GUI.
4. The GUI has a live preview mode that displays identification of points under various threshold settings. Using the configuration shown in Fig. 3, adjust the “Background param” slider until labels appear over each spot (Fig. 4b).

**Fig. 4:**
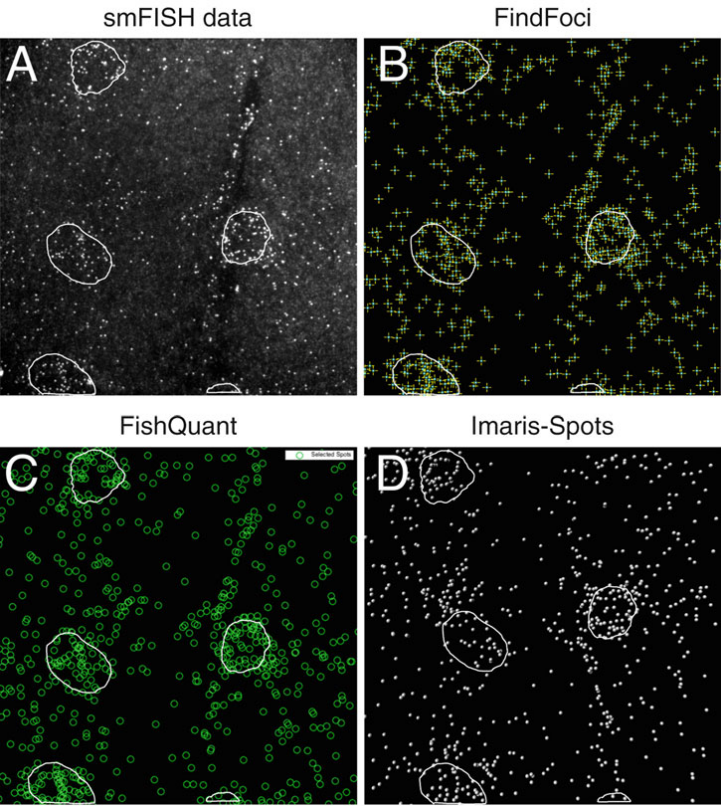
Quantification of transcript number using different spot counting applications. Each application has a GUI that displays which spots are detected as threshold parameters are adjusted. Nuclear regions, which can be segmented automatically with the DAPI channel, are shown here as circled regions. (**a**) Maximum projected stack of spinning disk confocal images showing MSP-300 smFISH sample. (**b**) Spots detected using the ImageJ FindFoci plugin. (**c**) Spots detected using the MatLab FishQuant software. (**d**) Spots detected using the Spots tool in Imaris
5. Number and features of the foci can be obtained from the measurement table or exported as a text file.

#### FISHQuant (Fig. 4c)

1. Save images to be analyzed into separate channels, FISH channel and marker channels for segmentation, and start the FISHQuant application in Matlab [17].
2. Follow the FISHQuant manual for loading data, filtering the image, and thresholding the spots.
3. Adjust the threshold parameters until each of the spots are marked in the GUI tool. The intensity profile will typically show an obvious separation between background and real spots (Fig. 3b).
4. Export the thresholded spots text file to determine the number of transcripts.

#### Imaris Spots (Fig. 4d)

1. Open the image with Imaris.
2. Choose the Spots tool.
3. Provide an estimated diameter for the spots (350 nm works well).
4. Slide the spot quality threshold tool until foci are accurately identified. The intensity profile will typically show an obvious separation between background signal and labeled mRNASingle molecule (Fig. 3b).
5. Save the statistics text file to determine the number of transcripts.

## Notes

1. Conventional confocal and widefield deconvolution microscopy techniques have a lateral resolution limit of ~250 nm and ~500 nm in the axial direction for green-emitting fluorochromes. 3D-SIM can enhance the lateral resolution to ~125 nm and axial resolution to ~250 nm. The effective measured diameter (full width half max) of a 1 kb folded mRNA molecule is ~150 nm in the red channel [28].
2. Fresh reagents (especially SSC and deionized formamide) are important for obtaining optimal signal-to-noise ratio. For best results, flash-freeze 1 mL aliquots of deionized formamide with liquid nitrogen and store at −80 °C. Reagents should be prepared with DEPC-treated water and autoclaved whenever possible.
3. It is important to correct for spherical aberration by matching the refractive index of the mountant with the immersion oil, or by adjusting the correction collar of the objective. 3D-SIM3D Structured Illumination Microscopy (3D-SIM) is very sensitive to artefacts caused by spherical aberration, particularly when imaging at depths greater than a few microns from the coverslip. While it is possible to correct spherical aberration to some extent when imaging deep with an oil immersion 1.42 NA objectives, we find the best results are obtained when imaging deep with a siliconeSingle molecule immersion objective, such as the 60× /1.3 from Olympus. Adaptive optics approaches hold the most promise for correcting aberration and remote focusing [29], but have not yet been popularized in off-the-shelf instruments.
4. Endogenous fluorescent proteins are well-preserved for confocal imaging but often bleach too quickly to acquire high quality SIM images. To overcome this problem, label fluorescent proteins with antibodies, such as the Chromotek lama anti-GFP antibody, coupled to a highly photo-stable Alexa Fluor or Atto dye of choice. The further the dye chosen emits into the red wavelengths, the better the signal–noise ratio because of reduced tissue autofluorescence, but the lower the resolution achievable, which is particularly important if 3D-SIM3D Structured Illumination Microscopy (3D-SIM) is used. The further the dye chosen emits into the red wavelengths the better the signal to background ratio because of reduced tissue autofluorescence, but the lower the resolution achievable, which IS particularly important if 3D-SIM is used.
5. To limit aberrations, the mounting medium must penetrate the tissue evenly so that the refractive index inside the cell matches the lens immersion oil, as much as possible.
6. After placing NMJ preparations on the glass slide, pipette 1 μL of 100 nm Tetraspek beads directly onto one of the preparations to use for aligning the different channels and for testing the quality of the point spread function (PSF).
7. All three software solutions support batch analysis. FindFoci performance is not as accurate as the others for data with low signal-to-noise. It is difficult to identify transcriptsSingle molecule in Imaris Spots with segmented regions of interest, e.g., the nucleus.
